# Deceleration capacity of heart rate variability as a predictor of sedation related hypotension

**DOI:** 10.1038/s41598-021-90342-z

**Published:** 2021-05-25

**Authors:** Feng-Fang Tsai, Chih-Min Liu, Hsiu-Po Wang, Jia-Rong Yeh, Shou-Zen Fan

**Affiliations:** 1 Department of Anaesthesiology, National Taiwan University Hospital, Taipei, Taiwan; 2 College of Medicine, National Taiwan University, Taipei, Taiwan; 3 Department of Research and Development, Shannon Investments Ltd., New Taipei City, Taiwan; 4 College of Medicine, National Taiwan University, Anesthesiology Office, 4th Floor, No.7.Chung San South Road, Taipei, 10002 Taiwan

**Keywords:** Cardiology, Mathematics and computing

## Abstract

High risk and geriatric patients are supposed to suffer higher risks of hypotension underwent painless endoscopic procedures. This study evaluated different biomarkers associated with hypotension in off-site patients and aimed to determine the most relevant risk factors in space and monitoring limited environment. The inclusions of this observational cohort study underwent complex endoscopic procedures were sedated with age-adjusted doses of target-controlled infusion of propofol. The following pre-sedative parameters were analysed: time domain, frequency domain, and Deceleration capacity (DC) of heart rate variability, estimated cardiac output data and the index of cardiac contractility from the cardiometer. Patients were divided into hypotension group (blood pressure < 90 mmHg or a > 35% decrease) and non-hypotension group according to peri-sedative blood pressure, regression analysis is used to examine the association between factors and hypotension. Total data from 178 patients (age range: 33–94 years) were analysed. Age was not significantly different between the hypotension and non-hypotension groups (*p* = 0.978). Among all the factors, DC was most associated with hypotension (*p* = 0.05), better than cardiometer, age, and ASA status. In conclusion, DC, which can be interpreted as the indicator of parasympathetic activity and was significantly and negatively correlated with sedation-related hypotension. Pre-sedative measuring DC from routine ECG monitoring is simple and cost-effective and should be added to haemodynamic monitoring in the endoscopic room.

## Introduction

Deceleration capacity (DC), derived from heart rate variability (HRV), represents the capacity of the cardiovascular system for dynamic changes while responding to physical and psychological stimuli. HRV is the physiological phenomenon of variation in the time interval between heartbeats; the time-domain HRV parameters include the standard deviation of Normal-to-Normal intervals (SDNN) and the root mean square power of the successive differences (RMSSD) between adjacent NN intervals. The low frequency/high frequency (LF/HF) ratio represents frequency-domain HRV^1^. Phase-rectified signal averaging (PRSA) is an efficient method for analysing quasi-periodicities in noisy and nonstationary time series, such as the HRV time series^2^. The central deflection of the PRSA curve characterises the average capacity of the heart to decelerate the cardiac rhythm, denoted as DC, which represents the ability of the parasympathetic nervous system to regulate cardiovascular modulation and thus serves as a functional biomarker for parasympathetic capacity. Evidence from experimental and clinical studies indicates that reduced DC is a better predictor of mortality than SDNN or left-ventricular ejection fraction in post-infarction patients^3,4^. DC and RMSSD of successive r–r intervals better represent parasympathetic modulation ability^5^. As a nonstationary time-series (such as a heartbeat time series), interruptions that might be caused by internal or external perturbations usually lead to phase jumps, and their frequency is not constant. Both phase jumps and frequency variations disrupt the auto-coherency of the signal. PRSA allows us to solve these issues in the quantification of the typical coherence time for each frequency band and to separate processes occurring during increasing and decreasing parts of the signal^6^.

Electrical cardiometry (EC) is a new noninvasively method of continuous cardiac output (CO) monitoring based on the measurement of thoracic electrical bioimpedance. The increasing velocity of red blood cell alignment in the thoracic aorta cause concomitant decreases in electrical impedance in the chest^7^. Compared with other portable noninvasive cardiometers, the EC monitor (ICON Cardiotronics, Inc.) has many benefits including low power, low cost, and convenient access to haemodynamic parameters^8^. It produces reliable data under various clinical conditions^9,10^ and estimates CO and the index of contractility (ICON), which are crucial physiological parameters for patient care^11^.

Invasive upper gastrointestinal endoscopic procedures require moderate to severe sedation instead of conscious sedation^12,13^ because the endoscopic insertion is painful (outer diameter, 13.7 mm). As the sedation level increases, patients lose the ability to protect the airway and sympathetic response to nociceptive stimulation^14^; other sedative-related complications can also occur^12,15,16^, including sedation-related hypotension. Because of the state of relative hypovolemia in patients after fasting and colon preparation^17^, combined with patient illness and diminution of the sympathetic effect due to sedation, hypotension often occurs, especially in elderly patients^18,19^. The short-term spasmolytic agent butylscopolamine (buscopan) can inhibit the parasympathetic regulation, leading to increased heart rate, thus decreasing homeostatic ability in elderly patients^20,21^. Propofol and opioids may cause vasodilation, which may deteriorate the already unpredictable perioperative haemodynamic of elderly patients^17,22^. Diabetes mellitus is a typical disease causing autonomic dysfunction, so clarification of the diseases that affect the autonomic nerves is important.

The aim of this study was to propose a reliable predictor of sedation-related hypotension for anaesthesiologists while performing sedation of off-site patients with limited space and restricted conditions. This research evaluated the risk of sedation-related hypotension due to age and compared related parameters to determine the most relevant factor predicting sedation-related hypotension.

## Results

### Demographic data

Twenty-two cases were missed because of incomplete physiological data. Table 1 presents the comparison of demographic data between the patients with and without hypotension (hypotension and no hypotension groups, respectively). The total incidence of hypotensive events was 42%. Sex and age were not significantly different between the two groups. The age range in the hypotension group was 33–94 years. No differences were observed in BMI and procedure time between the two groups; however, the hypotension group had a higher ASA class (*p* = 0.019). Also the HRV derivatives (DC, RMSD, HF/LF ratio) and EC derivatives (ICON, CO) was also compared. The t-test of ICON and CO showed that there were no differences between two groups (*p* = 0.165 and 0.925). The other analysis results of the hypotension group revealed the following: the mean DC was 4.68 ± 2.48, the mean RMSD was 26.2 ± 18.36; and the mean HF/LF ratio was 6.41 ± 1.68. The analysis results of non-hypotension group revealed the following: the mean DC was 6.16 ± 2.68 (*p* = 0.000); the mean RMSD was 32.9 ± 19.13 (*p* = 0.023); and the mean HF/LF ratio was 5.80 ± 1.88 (*p* = 0.03).

**Table 1:** Comparison of the clinical and demographic characteristics of 178 patients in the two groups using t test.

	Hypotension (n = 75)	No hypotension (= 103)	*p* Value
ERCP/ERBD/EUS	26/7/42	29/13/61	
Age; y	70.64 (12.9)	67.57 (12.9)	.978
Gender (M/F)	41/34	50/53	.424
BH ; cm	161.6 (7.6)	160.8 (8.2)	.459
BW; kg	60.7 (12.6)	60.0 (11.1)	.558
BMI	23.1 (3.9)	23.2 (3.8)	.438
ASA I/II/III	0/41/34	0/67/36	.019*
Bed ridden, n (%)	4 (5.3)	3 (2.9)	.103
CVA, n (%)	2 (2.7)	1 (1.0)	.084
HD, n (%)	2 (2.7)	1 (1.0)	.084
HTN, n (%)	45 (60)	53 (51.5)	.049*
Cancer, n (%)	39 (52)	43 (41.7)	.186
BW loss > 5%, n (%)	30 (40)	39 (37.9)	.574
Duration (min)	32.3 (17.5)	26.2 (15.1)	.555
ALT (U L^−1^)	57.5 (79.5)	60.1 (116.3)	.408
AST (U L^−1^)	61.4 (98.4)	46.1 (82.8)	.135
Albumin (g dL^−1^)	3.0 (1.7)	2.8 (1.9)	.008*
Hb (g dL^−1^)	12.6 (2.0)	12.7 (1.9)	.451
DC	4.68(2.48)	6.16(2.68)	.000
ICON	45.90 (17.48)	49.63(17.03)	.165
CO	4.54 (1.71)	4.51(1.54)	.925
RMSD	26.2(18.36)	32.9(19.13)	.023
LF/HF ratio	6.41(1.68)	5.80(1.88)	.030

Values are mean (SD) or number (proportion).

BH = body height, BW = body weight, BMI = body mass index, ASA = classification of the American Society of Anaesthesiologists, CVA = cerebral vascular accident, HD = haemodialysis, HTN = hypertension history, ALT = alanine aminotransferase, AST = aspartate aminotransferase, Hb = haemoglobin. *p < 0.05. DC = deceleration capacity, RMSD = root mean square density, ICON = index of contractility, LF/HF ratio = low frequency/high frequency ratio.

The history of chronic or systemic diseases was also compared between the groups. Of all the conditions evaluated, only hypertension was related to hypotensive events (*p* = 0.049). Other conditions, including bedridden status, cerebrovascular accident, haemodialysis (HD), cancer, and > 5% loss of bodyweight, were equivalent between the two groups. In the biochemical examination, albumin level, which represents the blood oncotic pressure, was significantly different between groups. ALT and AST showed large fluctuations between inclusions, are not related between groups. Haemoglobin level was not significantly different between groups. The total number of patients with type 2 DM was 34 out of 178, the number of patients with DM and hypotensive event was 17 out of 74 (23.0%), and the number of patients with DM in control group (normal pressure) was 17 out of 101 (16.8%). The univariate analysis of DM showed that there was no difference in hypotension events (*p* = 0.313). The other analysis results of the DM group revealed the following: the mean DC was 4.95 ± 2.71, the mean RMSD was 33.88 ± 24.29; the ICON was 41.63 ± 14.65, and the mean AC was 5.60 ± 3.05. The analysis results of non-DM group revealed the following: the mean DC was 5.68 ± 2.68 (*p* = 0.743); the mean RMSD was 29.07 ± 17.48 (*p* = 0.092); the ICON was 49.66 ± 17.55 (*p* = 0.103); and the mean of AC was 5.95 ± 2.97 (*p* = 0.826). The results of the DM group comparison are showed in Table 2.

**Table 2:** Comparison of the demographic characteristics of the DM group with non-DM group using t test.

	DM(34)	Non-DM(144)	Significance
Event (N)	50% (17)	40% (57)	.313
DC	4.95 ± 2.71	5.68 ± 2.68	.743
RMSD	33.88 ± 24.29	29.07 ± 17.48	.092
ICON	41.63 ± 14.65	49.66 ± 17.55	.103
LF/HF ratio	6.05 ± 1.5	6.05 ± 1.89	.296

Values are mean (SD) or number (proportion).

Event = hypotension was either systolic blood pressure < 90 mmHg^12^ or a decrease in mean blood pressure of > 35% during the first 10 min, DC = deceleration capacity, RMSD = root mean square density, ICON = index of contractility, LF/HF ratio = low frequency/high frequency ratio.

### Risk factor analysis

**Figure 1:**
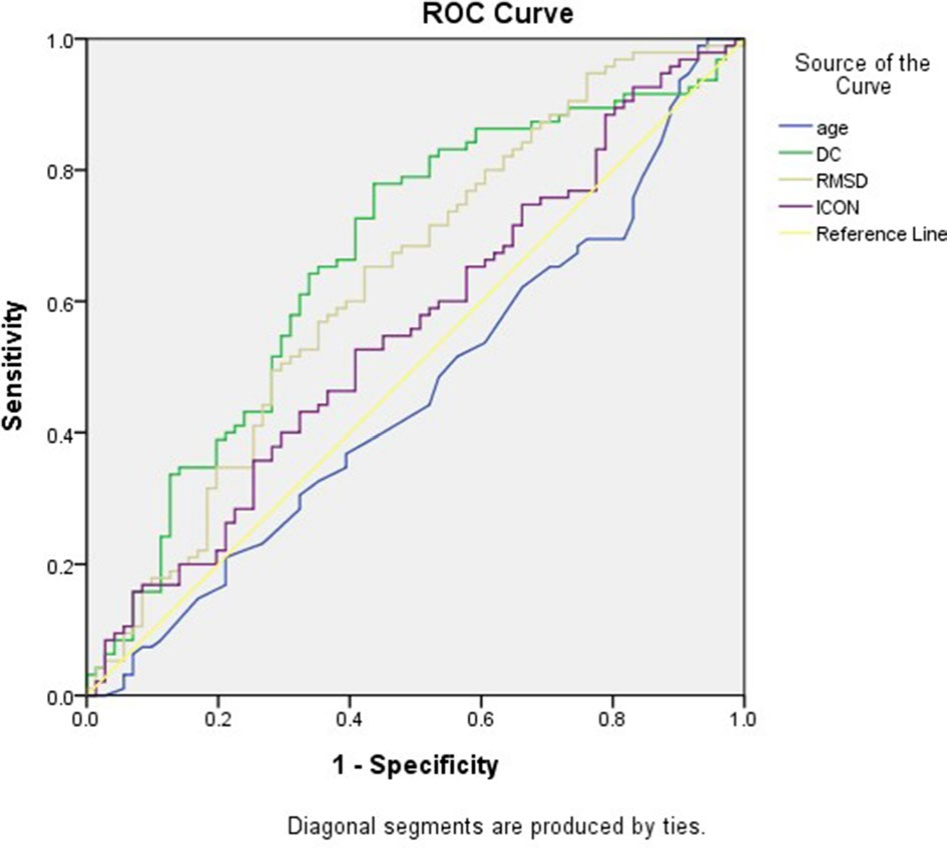
Receiver operating characteristic (ROC) curve for the factors predicting hypotension. The area under the curve (AUC) for deceleration capacity (DC) for the prediction of hypotension is 0.7 ± 0.05. The ideal AUC is 1.0. The reference line (yellow) represents an AUC of 0.5, which is based on chance alone. DC = deceleration capacity, RMSD = root mean square density, ICON = index of contractility.

Table 3 presents the results logistic regression analysis of factors related to hypotension. We used logistic regression to examine patients’ DC, RMSD, LF/HF ratio, from HRV; ICON and CO from EC, and the factors ASA, HTN and albumin which showed significant differences, as listed in Table 1. The beta coefficient value of DC was negatively related to hypotension; this means the lower the DC, the higher the risk of hypotension. No other variable significantly predicted the outcome of hypotension. As shown in Fig. 1, the area under the curve (AUC) for predicting hypotension was 0.7 ± 0.05, 0.6, 0.56, and 0.46 for DC, RMSD, ICON, and age, respectively. According the DC data, the minimum DC is 0.62, the maximum is 13.15, with mean 5.54 and SD 2,697; according to the result of logistic regression, DC was negatively related to outcome, the DC decrease 1 will increase the hypotension incidence 19%, the cut-off value would be 5.15.

**Table 3:** Results of the logistic regression analysis with hypotension (dependent) and potential risk factors.

		B	S.E	Wald	Sig	Exp(B)
Factors	DC	− .147	.076	3.775	.050	.863
	RMSD	− .018	.011	2.912	.088	.982
	LF/HF	.039	.104	.143	.706	1.040
	Albumin	.009	.095	.008	.928	1.009
	HTN	− .060	.353	.029	.866	.942
	ASA	.302	.372	.658	.417	1.352
	ICON	− .011	.010	1.188	.276	.989

DC = deceleration capacity, RMSD = root mean square density, LF/HF = low frequency/high frequency ratio, HTN = hypertension, ASA = classification of the American Society of Anesthesiologists, ICON = index of contractility.

## Discussion

Our results reveal that DC was the best independent predictor of sedation-related hypotension in patients undergoing an endoscopic procedure compared with other parameters of HRV, bioimpedance, or biochemistry; DC was negatively correlated with hypotension. We also used the ROC curves (Fig. 1) to reveal the differences between age, DC, RMSD, and ICON. DC, with an AUC of 0.7, was the most correlated predictor, with acceptable discrimination. We concluded that DC is a reliable predictor of sedation-related hypotension for anaesthesiologists while administering sedation in off-site patients under limited space and restricted conditions. If the DC data can be shown on the haemodynamic monitor along with ECG data, it would be providing convenient and useful information for anaesthesiologists.

Between the hypotension and non-hypotension groups, the basic clinical and demographic data including age, sex, BMI, and procedure time were not significantly different. Age does not represent physiological ageing, and a patient should be evaluated sedative risk according to his or her organ function. No significant differences were observed in past or present history items such as cancer, renal failure, and CVA, except hypertension. In the biochemistry examination, low albumin level was related to hypotension due to low oncotic pressure; the ASA class was also significantly different between groups. This shows that a higher ASA class and lower oncotic pressure is related to hypotension, which is compatible with the risk factors of perioperative hypotension. After the data survey, inclusions with a history of DM had larger incidence of hypotension during sedation without significance. This is because diabetes mellitus with poor control may causes systemic deterioration, including autonomic dysfunction and may not be suitable for the protocol of this study. Anesthesiologists may prefer to use other hypnotics for those patients.

There are two major artefacts in bioimpedance cardiographs: motion artefacts and arrhythmia. During data collection, well-controlled conditions are required to obtain accurate and reliable measurements, which is time consuming. Each time the patient changes position, it takes approximately 2 min to recalculate the parameters. The leads are placed at the left side of patient, and they may have no signal in the decubitus position due to the increased resistance offered by the skin fold. Moreover, the bioimpedance monitor cannot calculate data when a patient has arrhythmia, which is common in elderly patients. In this study, the bioimpedance monitor was not convenient to use or suitable for off-site anaesthesia. Neither EC nor electrocardiography can be used to calculate cardiac biomarkers in patients who have arrhythmia, so we skipped the arrhythmia episode. We waited until the patient has normal sinus rhythm to obtain the first calculable data.

Our patients received invasive procedures with ERCP, EUS, or ERBD. For these procedures, not only is the outer endoscopic diameter larger (13.7 mm) but also the procedure is longer and more invasive; moreover, it is often performed in patients who are weaker. In contrast to panendoscopy, our anaesthetic regimens include opioid, midazolam, and propofol. When the patient is heavily sedated, irregular respiratory pattern and profound vasodilation might occur; sometimes, partial upper airway obstruction is observed, which requires a chin-lift manoeuvre or nasal airway insertion for a while. In our cohort, the overall hypotension incidence during sedation was 42%, which reverted to within the normal range in all cases after initial induction. The age-adjusted dosage maintained with a TCI was convenient for sedation during the endoscopic procedure.

According to the results, the cut-off point of DC is 5.15, patients who need invasive endoscopic procedure with DC less than 5.15 could inform anaesthesiologist for poetical hypotension, whether the patient requires an alternative sedative regimen or volume expansion before sedation. In conclusion, DC was negatively correlated with sedation-related hypotension, and age was not a predictor of sedation-related hypotension.

## Methods

### Ethics statement

This observational study was approved by the Institutional Review Board of National Taiwan University Hospital with registered number 201612030RIN and was also registered on ClinicalTrials.gov with registered number NCT03292627 (date of registration 25/09/2017). All participants provided written informed consent after careful discussion. We confirmed that all methods were carried out in accordance with relevant guidelines and regulations.

### Clinical inclusion

We included 200 patients who underwent endoscopic retrograde cholangiopancreatography (ERCP), an endoscopic ultrasound-guided procedure (EUS), or endoscopic retrograde biliary drainage (ERBD) between March 2017 and July 2019 in National Taiwan University Hospital.

**Figure 2:**
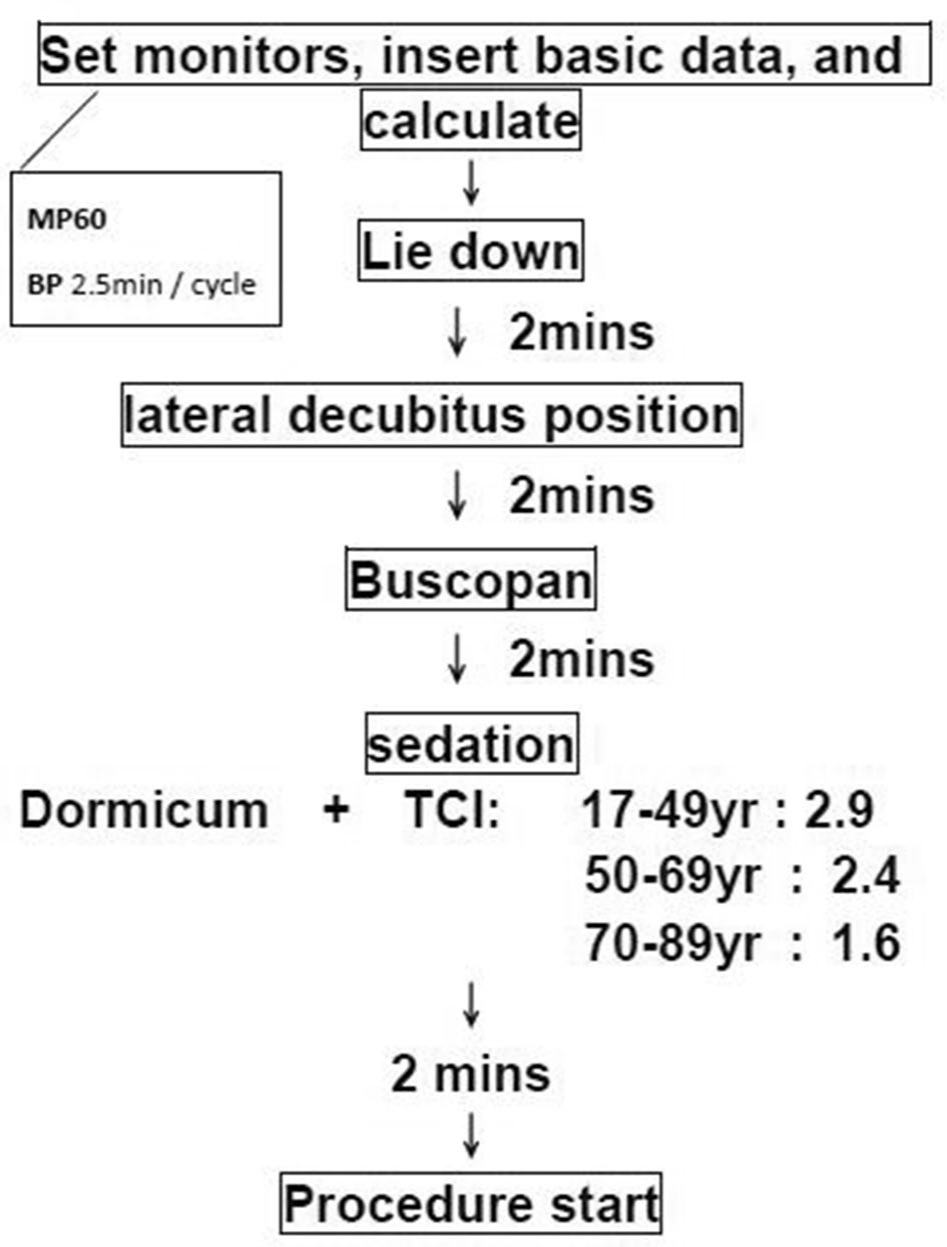
Flowchart of clinical protocol.

Prior to endoscopic insertion, the standard equipment for vital signs was employed: ECG, a blood pressure cuff used with a 2.5-min inflation interval, and a nasal cannula with 3 L min^−1^ oxygen. An EC monitor (ICON Cardiotronics, Inc., La Jolla, CA 92,307; Osypka Medical GmbH, Berlin, Germany) was set up, with electrodes placed around the neck and laterally on the thorax as well. The sampling rate of this machine was 1 Hz, and it calculated the patient’s cardiac output data and ICON value every second, which was displayed on the screen. Our data collection can only record inclusions who can have cardiac bioimpedance data; if the patient had arrhythmia such as atrial fibrillation or frequent ventricular premature complex so that the physiological cardiac parameter could not be calculated, it was marked as missing data. Butylscopolamine (buscopan) was administered after the patient was well postured, and 2 min later, sedation was initiated using midazolam (dormicum) (0.025 mg kg^−1^) and alfentanil (5 ng kg^−1^) combined with propofol infusion through a target control infuser (TCI). The TCI settings (Schneider mode) were as follows: plasma propofol concentration for patients < 50 years old was 2.9 ng mL^−1^, that for patients 50–70 years old was 2.4 ng mL^−1^, and for patients > 70 years old it was 1.6 ng mL^−1^^23^. The endoscopic procedure started 2 min after propofol infusion, and the investigators collected all the clinical hemodynamic data including NBP, HR and saturation until the end of the procedure (Fig. 2). Data related to body weight loss of > 5%, ambulation capacity, systemic disease, patient demographics, and biochemistry results were also collected.

### Data analysis

**Figure 3:**
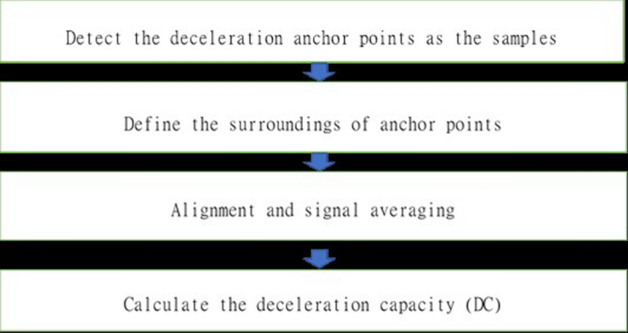
Flowchart of DC processing, further biophysics calculation equations are in the supplement.

The original electrocardiogram data were analysed using a commercial software package (MATLAB for Windows Ver4.2) for HRV profile. Pre-sedative two minute electrocardiogram data were analyzed. For the peri-sedative hemodynamic data, the criteria for hypotension were either systolic blood pressure < 90 mmHg^12^ or a decrease in mean blood pressure of > 35% during the first 10 min of the endoscopic procedure^22^. The fundamental feature of phase-rectified signal averaging is the alignment of the tachogram of HRV relative to selected anchor points followed by signal averaging. PRSA involves four main steps as followed Fig. 3^2^.

### Statistical analysis

We compared the supine baseline heart rate variability data with clinical hemodynamic data during sedation. SPSS (IBM SPSS Statistics Data Editor version 20) was used for data analysis. Demographic data, including age, height, weight, and presence of cancer, were compared using an independent t test. We also divided the breakdown of these diseases into a hypotensive patient group and a nonhypotensive patient group and summarized them in a table. Logistic regression was used to evaluate the relationship of risk factors (predictors), we choose potential candidate including DC, ICON, RMSD, HF/LF ratio, ASA, HTN and Albumin with hypotension (dependent variable). The *p* value less than 0.05 was set as significant.

## Supplementary Information

Supplementary Information.
