# Supplementary material for: Deceleration capacity of heart rate variability as a predictor of sedation related hypotension
Source: Sci Rep. 2021 May 25;11:10850. doi: 10.1038/s41598-021-90342-z (PMC8149708; doi:10.1038/s41598-021-90342-z)
Supplement: Supplementary file 1 — Supplementary Information. [file 41598_2021_90342_MOESM1_ESM.docx]

Deceleration capacity of heart rate variability as a predictor of sedation related hypotension

Feng-Fang Tsai^1^, Chih-Min Liu^2^, Hsiu-Po Wang^3^, Jia-Rong Yeh^4^, Shou-Zen Fan*^5^

1 Attending, Department of Anaesthesiology, National Taiwan University Hospital, Taipei, Taiwan. 2 Attending, Department of Anaesthesiology, National Taiwan University Hospital, Taipei, Taiwan. 3 Professor of Internal Medicine, College of Medicine, National Taiwan University, Taipei, Taiwan. 4Research Centre for Adaptive Data Analysis, National Central University, Taoyuan, Taiwan. 5 Associated Professor of Anaesthesiology, College of Medicine, National Taiwan University, Taipei, Taiwan

Description of Phase-Rectiﬁed Signal Averaging

Alice Feng-Fang Tsai, Jia-Rong Yeh

Detect the deceleration anchor points as the samples $x_{i_{v}}$, which satisfying the condition of $x_{i_{v}}> x_{i_{v}-1}$

Extract the surroundings of anchor points: a four samples segment [$x_{i_{v}-1}$ $x_{i_{v}}$ $x_{i_{v}+1}$ $x_{i_{v}+2}$] is defined as the surrounding of $x_{i_{v}}$ for $v=1,\ldots,M$

Alignment and signal averaging: align all surroundings by anchor points and obtain the averaged four-elements segment of all surroundings [$\bar{x}_{-1}$ $\bar{x}_{0}$ $\bar{x}_{1}$ $\bar{x}_{2}$], in which $\bar{x}_{k}=\frac{1}{M}\sum_{v=1}^{M} x_{i_{v}+k}$ and $\bar{x}_{0}$ is the average of anchor points

Calculate the deceleration capacity (DC):

$$DC=\frac{\bar{x}_{2}+\bar{x}_{1}-\bar{x}_{0}-\bar{x}_{-1}}{4}$$

The fundamental of the phase-rectiﬁed signal averaging is the alignment of windows of the series relative to selected anchor points followed by a signal averaging. In PRSA, there are four main steps as shown in the followings:

1. Detect the anchor points

Anchor points are picked up from signal (*x_i_*) according to a certain property. The most common properties used in PRSA are increase or decrease events. The anchor points corresponding to increases in signal are defined as the samples satisfying the condition of *x_i_ > x_i-1_*. Alternatively, the anchor points corresponding to decreases in signal are selected as the samples satisfying the condition of *x_i_ < x_i-1_*. In PRSA, we pickup all anchor points under increase or decrease condition. Large increases or decreases, with changing rate > 5%, are excluded from selected anchor points. Such increases or decreases are often related to ectopic beats or measurement artifacts.

1. Define the surroundings

In PRSA, surroundings with window size of 2L, here L is the wing length of surrounding, are defined around each anchor points. Anchor points with full surroundings are used in this study. If the positions of all regarded anchor points are denoted as *i_v_*, *v = 1, …, M*, the points of surrounding number *v*, corresponding to anchor point *i_v_,* will be

$x_{i_{v}-L}, x_{i_{v}-L+1},\ldots,x_{i_{v}},\ldots,x_{i_{v}+L-2},x_{i_{v}+L-1}$ (1)

There, *M* is the number of anchor points and their surroundings.

1. Signal averaging

The phase-rectiﬁed signal average $\bar{x}_{k}$ is obtained by averaging over all M surroundings,

$\bar{x}_{k}=\frac{1}{M}\sum_{v=1}^{M} x_{i_{v}+k}$, for *k = -L, …, 0, ..., L-1* （2）

1. Calculate the deceleration or acceleration capacity

We defined the deceleration capacity (DC) for a human heart beat time series by the phase-rectified signal average $\bar{x}_{k}$ using anchor points of increases. The DC can be determined as

$DC=\frac{\bar{x}_{2}+\bar{x}_{1}-\bar{x}_{0}-\bar{x}_{-1}}{4}$ （3）

Alternatively, acceleration capacity (AC) can be calculated by equation (3) from the phase-rectified signal average $\bar{x}_{k}$ using anchor points of decreases.
